# Development and validation of a novel scale for antiretroviral therapy readiness among pregnant women in urban Zambia with newly diagnosed HIV infection

**DOI:** 10.1186/s12981-023-00509-z

**Published:** 2023-04-06

**Authors:** Mwangelwa Mubiana-Mbewe, Samuel Bosomprah, Rakesh Kumar Saroj, Jillian Kadota, Aybuke Koyuncu, Kusanthan Thankian, Michael J. Vinikoor

**Affiliations:** 1 Centre for Infectious Diseases Research in Zambia, Plot 34620 Off Alick Nkhata Road, P.O. Box 34681, Lusaka, Zambia; 2 Department of Biostatistics, School of Public Health, University of Ghana, Accra, Ghana; 3 School of Computational and Integrative Sciences, Jawaharlal Nehru University, New Delhi, 110067 India; 4 UCSF Center for Tuberculosis and Division of Pulmonary and Critical Care Medicine San Francisco General Hospital, University of California, San Francisco, CA USA; 5 Department of Epidemiology, Johns Hopkins University, Maryland, USA; 6 Department of Gender Studies, University of Zambia, Lusaka, Zambia; 7 Department of Medicine, University of Alabama at Birmingham, Birmingham, USA

**Keywords:** HIV, Pregnant women, ART readiness, Option B +, ART initiation

## Abstract

**Background:**

Women who are newly diagnosed with HIV infection during pregnancy may not be ready to immediately initiate lifelong antiretroviral therapy (ART; called Option B +) as is recommended. Lack of “readiness” drives early disengagement from care and undermines prevention of HIV transmission to infants. Several studies have shown high early attrition of women initiating ART in pregnancy. Although poor ART uptake and adherence have been attributed to various factors including stigma, disclosure issues and structural issues, there is no standard way of determining which pregnant woman will face challenges and therefore need additional support. We developed and validated a novel ART readiness tool in Lusaka, Zambia.

**Methods:**

The aim of this study was to develop and validate a tool that could be used to assess how ready a newly diagnosed pregnant woman living with HIV would be to initiate ART on the day of diagnosis. Using a mixed method design, we conducted this study in three public-setting health facilities in Lusaka, Zambia. Informed by qualitative research and literature review, we identified 27 candidate items. We assessed content validity using expert and target population judgment approaches. We administered the 27-item questionnaire to 454 newly diagnosed pregnant women living with HIV, who were enrolled into a randomized trial (trials number NCT02459678). We performed item reduction analysis and used Cronbach’s alpha coefficient of 0.70 as threshold for reliability.

**Results:**

A total of 454 pregnant women living with HIV enrolled in the study between March 2017 and December 2017; 452 had complete data for analysis. The correlation coefficient between the 27 items on the completed ART readiness scale ranged from 0.31 to 0.70 while item discrimination index ranged from -0.01 to 2.38. Sixteen items were selected for the final scale, representing three domains, which we classified as “internalized and anticipated HIV stigma”, “partner support” and “anticipated structural barriers”.

**Conclusion:**

We developed and validated a tool that could be used to assess readiness of newly diagnosed women living with HIV to initiate ART. This ART readiness tool could allow clinics to tailor limited resources to pregnant women living with HIV needing additional support to initiate and remain on ART.

**Supplementary Information:**

The online version contains supplementary material available at 10.1186/s12981-023-00509-z.

## Background

The pediatric Human Immunodeficiency Virus (HIV) epidemic remains a challenge with an estimated 1.7 million children living with HIV globally at the end of 2020, mostly in sub-Saharan Africa [1]. Majority of these children acquire HIV from their mothers during pregnancy, birth or breastfeeding [2]. Despite the successes of the Prevention of Mother to Child Transmission of HIV (PMTCT) program in Zambia, with an estimated 12,000 averted pediatric HIV infections, there were an estimated 6000 new HIV infections in children aged 0-14 years in 2020; about 90% of whom acquired the disease through mother to child transmission [3]. HIV prevalence in pregnancy has remained high at 10.3% [4]. In 2012, the world health organization (WHO) recommended rapid initiation of lifelong antiretroviral therapy (ART) to all pregnant and breastfeeding women with HIV irrespective of CD4 count and/or WHO stage, known as option B +  [5] because of its health benefit and simplification of logistics [6]. This approach was adopted in Zambia beginning in February 2013 [7].

Despite the purported health benefits of Option B + for both mother and infant, some women may not be ready to start lifelong ART during pregnancy or breastfeeding, particularly if they perceive themselves to be relatively healthy [8–10]. Years after implementation of option B + , same day acceptability remains suboptimal. Our earlier data found same-day ART initiation to be 74.7%, rising to 92.1% by 7 days [11]. A study done in a similar setting found that 77.8% of women living with HIV reported willingness to accept life-long ART during pregnancy or postnatally [10]. Barriers are centered around partner support and disclosure issues [12]. In the pre-Option B + era, several sessions of counseling, over weeks, were routinely provided to ‘prepare’ patients for long-term ART; but the focus at present is ART start on the day of HIV diagnosis or as soon as possible. This raises concern for initiation of and adherence to lifelong treatment in pregnant women living with HIV, especially when Option B + aims to cover women who may perceive themselves to be relatively healthy. In fact, early attrition on ART, particularly after the first dispensation of drug is common and has become a major focus area for PMTCT programs [13, 14]. Identifying those women who are not ready to start ART immediately and providing them with targeted ART adherence and retention support, may be an efficient approach for programs to optimize Option B + .

In this study, we describe the development and validation of an Option B + Readiness tool in Zambia. This builds on similar tools, including the HIV Medication Readiness Scale, a 10-question survey in Canadian study population [15], and the 49-item HIV Treatment Readiness Measure, which was designed for adolescents in the U.S. [16]. Nested with a randomized control trial of an Option B + enhanced adherence intervention, we explored the construct of ‘readiness’ and developed a tool for its measurement. We sought to develop a pragmatic instrument that could help antenatal care (ANC) programs to identify in advance the women with HIV who are at high risk of early attrition from the PMTCT cascade and would allow targeted interventions to be made for these women. We hope that this tool can be useful in resource-limited settings in assessing readiness of pregnant women living with HIV to initiate and adhere to ART.

## Methods

### Study settings

We implemented a 3-phased study comprising of (1) formative research exploring the issues of patient readiness to initiate lifelong ART among pregnant women with HIV, as well as ART adherence and retention in HIV care and treatment during pregnancy, (2) creation and validation of a readiness assessment tool and enhanced adherence package and (3) a randomized trial assessing the effectiveness of the enhanced adherence package (National Clinical Trials (NCT) number, 02459678). The study was implemented at 3 large volume urban public-sector health facilities in Lusaka District namely: Kanyama 1st Level Hospital, Matero 1st Level Hospital, and Chawama 1st Level Hospital. Within the maternal and child health departments, newly diagnosed pregnant women living with HIV were recruited to the clinical trial. Participants were included if (a) 18 years and older, (b) ART-naïve, and (c) became eligible to start ART under the Option B + approach within the past 7 days. Women with known intrauterine fetal demise, known history of mental illness and those who did not intend to receive antenatal care, labour and delivery at the study health facilities were excluded. After written informed consent, women were randomized to a B + enhanced adherence support intervention or the standard of care. As part of baseline procedures, an ART Readiness tool, created from phase 2 of the study was completed. We now further describe the development of the readiness scale.

### Item development

We used both inductive and deductive approaches to identify the item pool [17, 18]. Regarding the deductive approach, we reviewed the literature to identify barriers and facilitators to ART initiation, ART adherence, and retention in care [19–26]. We have also assessed existing scales and indicators including the HIV Medication Readiness Scale [15] and HIV Treatment Readiness Measure [16].

Regarding the inductive approach (study phase 1), we used formative qualitative research methodologies including in-depth interviews (IDI) with 24 pregnant and breastfeeding women living with HIV and focus group discussions (FGD) with 16 male partners to explore patient readiness, and barriers and facilitators to initiate and adhere to lifelong ART among pregnant women with HIV. The IDI and FGD facilitators were independent qualitative researchers who were not part of the care team for the participants and were supervised by TK. We conducted the IDIs and FGDs in one of two local languages (Bemba or Nyanja) or English. All the IDIs and FGDs were recorded and then transcribed verbatim if conducted in English. IDIs and FGDs that were conducted in a local language were first translated by the interviewer and back-translated by someone who did not conduct the IDI or FGD for quality assurance. Topics of discussion included those related to initiation and adherence to medication and appointments, such as attitudes, emotions, social support, transportation, HIV-related stigma, and financial costs among others. Using Atlas qualitative software, we performed thematic content analysis. This was done in the following five steps. First, reading for content: we began with data reading until content becomes intimately familiar. As data were reviewed, emergent themes were noted. Topics that previous research has not adequately addressed and ones that emerge unexpectedly were explored in continued fieldwork. Second, coding: a list of codes was created based on identified themes and assigned to specific sections of text so that the text was easily searched. Code definitions were documented in a code book. Third, data reduction: once transcripts have been coded, we worked within each code to identify principal sub-themes that reflected finer distinctions in the data. Fourth, data display: matrices and tables that categorized and displayed data were used to help facilitate comparisons. Fifth, interpretation: once text had been read and coded, and central ideas extracted, we identified and explained the core meanings of the data.

The combined approaches generated a 27-item pool as shown in Table 1 on page 9. We conducted content validity assessment using expert and target population judgment approaches (study phase 2). First, we identified experts who are knowledgeable about HIV but who have not been involved in the item pool development. They provided comments on the item pool regarding its relevance to the ART readiness construct. Based on feedback from these experts, we decided to proceed with a 3-level response (rather than the desirable 5-levels), which was felt to be more practical for at-scale use of the tool. Second, we conducted 3 FGD with 24 pregnant women living with HIV on or eligible for ART and 3 FGD with 24 health care workers for content validity (8 in each group); items were reviewed for clarity. This was to ensure that the items capture the relevant experience of the targeted pregnant women living with HIV.

**Table 1:** Initial items of the ART readiness scale for pregnant women living with HIV

Item code	Items	Response format		
		Disagree (no)	Not sure	Agree (yes)
q1	Do you plan to tell most people about your HIV status?	0	1	2
q2	Do you believe that telling someone you have HIV is risky?	0	1	2
q3	I feel guilty that I am HIV positive	0	1	2
q4	Some people who learn my HIV status will start avoiding me	0	1	2
q5	Some people will tell me that it's my fault I have HIV	0	1	2
q6	I fear that people who know that I have HIV will tell others	0	1	2
q7	I worry that people may judge me when they learn my HIV status	0	1	2
q8	Most people with HIV are rejected when others learn their status	0	1	2
q9	If he knew my HIV status, my partner would help me with taking ARVs	2	1	0
q10	If he knew my HIV status, my partner would leave me or chase me from the house	0	1	2
q11	My partner cares about me and take care of me	2	1	0
q12	My partner would escort me to the clinic if I asked	2	1	0
q13	Would knowing your partner's status encourage you to take your ARVs?	2	1	0
q14	When times were hard in the past, I felt supported and encouraged by my family, friends, neighbours, or church	2	1	0
q15	I have a close relative or friend i can talk to about my ARVs	2	1	0
q16	It is okay to stop taking ARVs once you feel better	0	1	2
q17	It is okay to stop taking ARVs after baby is born	0	1	2
q18	I know that I will be able to take all my ARVs correctly	2	1	0
q19	I am afraid that ARVs will cause serious side effects	0	1	2
q20	Taking ARVs would not really help me	0	1	2
q21	Do you believe that prayers alone can cure you of HIV?	0	1	2
q22	Do you believe traditional medicines would be better than ARVs to treat or cure HIV?	0	1	2
q23	I fear that i may not always have enough food to take with my ARVs	0	1	2
q24	The cost of transportation to clinic may keep me from picking up my refills of ARVs on time	0	1	2
q25	It will be hard to find time to come to clinic to pick up my ARVs	0	1	2
q26	The lines at the clinic are too long	0	1	2
q27	Do you believe that some of the staff at the clinic are not friendly?	0	1	2

### Scale development

***Pre-testing and main survey***: Before the trial, we also pretested the questions in 25 HIV-positive pregnant women in one other clinic. This was to ensure that items are meaningful to the target population before the survey is administered. It also helped us to eliminate poorly worded items and facilitated rephrasing of items. As mentioned, the initial 27-item ART readiness scale was administered to all trial participants and four hundred and fifty-two (452) women completed it. This sample size was well above the rule of thumb of at least 10 participants per scale item and allowed for item reduction procedures, which requires bootstrapping [27, 28]. The readiness scale was administered in one of the two local languages (Bemba or Nyanja) or English by research team members who were conversant with all three languages.

We recoded all items into a binary response scale so that an item takes a value of 1 if the response has positive correlation with readiness and 0 otherwise. We used tetrachoric correlation coefficient to assess the correlation between items. Items with low correlation coefficient (< 0.3) are less desirable and were excluded from the item pool. We used Item Response Theory (IRT) to estimate discrimination index. Items with discrimination index having a p-value > 0.05 were further excluded [29, 30].

We used exploratory factor analysis (EFA) to extract factors and to assess the contribution of the items to the construct using rotated factor loadings. We used eigenvalue greater than 1 [31] to determine the number of factors extracted. Items with orthogonal rotated factor loadings less than 0.40 were further excluded [27, 32]. Also, items with cross-loadings or that appear not to load uniquely on individual factors were deleted. We used Kaiser–Meyer–Olkin (KMO) measure of sampling adequacy to assess whether there is enough covariation in the variables to allow for us to extract the factors. A KMO greater or equal to 0.6 was considered adequate. We also used anti-image correlation and covariance matrices to assess whether there is any correlation between the variable itself and any underlying factors. Any variable with a variance < 0.5 is excluded in the factor analysis. All analyses were performed using Stata 15 MP (StataCorp, College Station, TX, USA).

### Scale evaluation

We did not perform confirmatory factor analysis to test the dimensionality of the hypothesised factors extracted from the EFA. This is because we did not have a test dataset from either data collected at a different time point in a longitudinal study or a new dataset. We used Cronbach’s alpha to assess the internal consistency of the scale items, in terms of the degree to which the set of items in the scale co-vary, relative to their sum score [31, 33]. We considered an alpha coefficient of 0.70 as an acceptable threshold for reliability.

## Results

### Characteristics of participants

A total of 454 pregnant women living with HIV enrolled in the study between March 2017 and December 2017. Median age was 26.9 years (Interquartile range; IQR 23.6–31.8); median gestational age was 22 weeks (IQR 18–25). Two participants had missing data on the readiness tool, leaving 452 participants available for further analysis. Characteristics of the enrolled women are shown in Table 2 on page 12.

**Table 2:** Baseline descriptive characteristics of enrolled participants (N = 454)

Characteristics at baseline	Study arm		p-value
	BEAP (n = 229)	Standard-of-care (n = 225)	
Age, years	26.3 (23.3–31.7)	27.5 (24.0–32.0)	0.16
Gestational age, weeks	22 (18–25)	23 (19–26)	0.09
Number of Pregnancies	3 (2–4)	3 (2–4)	0.23
Number of live births	2 (1–3)	2 (1–3)	0.37
Marital status			0.48
Cohabitating/Married	200 (87.3)	199 (88.4)	
Single/Widowed/Divorced	29 (12.7)	26 (11.6)	
Educational level			0.28
None	15 (6.6)	11 (4.9)	
Nursery/Primary	69 (30.1)	87 (38.7)	
> = Secondary	145 (63.3)	127 (56.4)	
Initiated ART on same day as diagnosis^a^	167 (72.9)	172 (76.8)	0.30
Initiated ART at/before enrollment	204 (89.1)	208 (92.4)	0.32
CD4 Count^b^	287 (195.5–392.5)	317 (201–426)	0.20
CD4 Count^b^			0.14
< 350 cells/mm3	113 (64.2)	99 (56.6)	
≥ 350 cells/mm3	63 (35.8)	76 (43.4)	
Clinic			1.00
Kanyama	76 (33.2)	75 (33.3)	
Chawama	77 (33.6)	75 (33.3)	
Matero	76 (33.2)	75 (33.3)	
Disclosure of HIV status to male partner at/before enrollment	84 (36.7)	73 (32.4)	0.22
Participant aware of male partner’s HIV status	77 (33.6)	64 (28.4)	0.28
Of those aware, whose partner is positive	39 (50.6)	40 (62.5)	0.25

Reprinted from “Effect of enhanced adherence package on early ART uptake among HIV-positive pregnant women in Zambia: an individual randomized controlled trial”, by Mubiana-Mbewe, M. AIDS and Behaviour 2021 Mar; 25(3):992–1000 [11]

^*^Data are in n (%) or Median (IQR) with Chi-Squared and Wilcoxon Rank-Sum p-values, respectively

^a^1 missing value (0.22%)

^b^103 missing values (23%)

### Indices

The correlation coefficient between the 27 items on the initial ART readiness scale ranged from 0.31 to 0.70. The item discrimination index ranged from -0.01 to 2.38 (Table 3 on page 13). Six items had discrimination index with p-value greater than 0.05 and were discarded as poorly discriminating questions. The results showed that sixteen items were functional to ART readiness construct with rotated factor loadings ranging from 0.387 to 0.679 (Table 4 on page 14). The scale derived from our items appears to be reasonable because the estimated correlation between it and the underlying factor it measures is $\sqrt{0.77}\approx 0.88$ and the estimated correlation between this battery of sixteen items and all other sixteen-item batteries from the same domain is 0.77 (Table 4 and Additioanl file 1: Table S1).

**Table 3:** Item discrimination index in ascending order

Item code	Items	Discrimination index	P-value*	95% CI	
q26	The lines at the clinic are too long	− 0.01	0.944	− 0.22	0.20
q27	Do you believe that some of the staff at the clinic are not friendly?	0.00	0.975	− 0.22	0.21
q18	I know that I will be able to take all my ARVs correctly	0.06	0.716	− 0.24	0.35
q16	It is okay to stop taking ARVs once you feel better	0.11	0.564	− 0.27	0.49
q17	It is okay to stop taking ARVs after baby is born	0.19	0.292	− 0.17	0.56
q22	Do you believe traditional medicines would be better than ARVs to treat or cure HIV?	0.33	0.148	− 0.12	0.77
q1	Do you plan to tell most people about your HIV status?	0.34	**0.014**	0.07	0.62
q15	I have a close relative or friend I can talk to about my ARVs	0.39	**0.002**	0.14	0.63
q21	Do you believe that prayers alone can cure you of HIV?	0.42	**0.003**	0.15	0.70
q14	When times were hard in the past, I felt supported and encouraged by my family, friends, neighbours, or church	0.43	**0.004**	0.14	0.72
q9	If he knew my HIV status, my partner would help me with taking ARVs	0.47	**0.000**	0.21	0.74
q20	Taking ARVs would not really help me	0.60	**0.001**	0.23	0.96
q25	It will be hard to find time to come to clinic to pick up my ARVs	0.64	**0.004**	0.21	1.06
q11	My partner cares about me and take care of me	0.70	**0.000**	0.33	1.06
q10	If he knew my HIV status, my partner would leave me or chase me from the house	0.71	**0.000**	0.45	0.97
q19	I am afraid that ARVs will cause serious side effects	0.73	**0.000**	0.46	1.00
q24	The cost of transportation to clinic may keep me from picking up my refills of ARVs on time	0.84	**0.000**	0.43	1.25
q12	My partner would escort me to the clinic if I asked	1.04	**0.000**	0.67	1.40
q13	Would knowing your partner's status encourage you to take your ARVs?	1.13	**0.000**	0.65	1.60
q2	Do you believe that telling someone you have HIV is risky?	1.16	**0.000**	0.84	1.48
q23	I fear that I may not always have enough food to take with my ARVs	1.34	**0.000**	0.92	1.76
q3	I feel guilty that I am HIV positive	1.54	**0.000**	1.12	1.96
q6	I fear that people who know that I have HIV will tell others	1.62	**0.000**	1.18	2.07
q8	Most people with HIV are rejected when others learn their status	2.02	**0.000**	1.50	2.55
q4	Some people who learn my HIV status will start avoiding me	2.12	**0.000**	1.57	2.67
q7	I worry that people may judge me when they learn my HIV status	2.12	**0.000**	1.57	2.67
q5	Some people will tell me that it's my fault I have HIV	2.38	**0.000**	1.74	3.02

*Items with *p*-value<0.05 (in bold)

**Table 4:** Validated antiretroviral therapy readiness scale for pregnant women living with HIV

Item code	Items	Factor loadings			Domain
		Factor 1	Factor 2	Factor 3	
q2	Do you believe that telling someone you have HIV is risky?	0.4757			Internalized and anticipated HIV stigma
q3	I feel guilty that I am HIV positive	0.435			
q4	Some people who learn my HIV status will start avoiding me	0.6618			
q5	Some people will tell me that it's my fault I have HIV	0.6787			
q6	I fear that people who know that I have HIV will tell others	0.5687			
q7	I worry that people may judge me when they learn my HIV status	0.6106			
q8	Most people with HIV are rejected when others learn their status	0.6166			
q9	If he knew my HIV status, my partner would help me with taking ARVs		0.5614		Partner support
q10	If he knew my HIV status, my partner would leave me or chase me from the house		0.4364		
q11	My partner cares about me and takes care of me		0.5996		
q12	My partner would escort me to the clinic if I asked		0.501		
q13	Would knowing your partner's status encourage you to take your ARVs?		0.5639		
q19	I am afraid that ARVs will cause serious side effects			0.4547	Anticipated structural barriers
q23	I fear that I may not always have enough food to take with my ARVs			0.3874	
q24	The cost of transportation to clinic may keep me from picking up my refills of ARVs on time			0.3914	
q25	It will be hard to find time to come to clinic to pick up my ARVs			0.4108	

The sixteen items loaded uniquely on three individual factors: factor 1 having 7 items, factor 2 having 5 items, and factor 3 having 4 items (Table 4). These three factors represent three domains, namely, “internalized and anticipated HIV stigma” (factor 1), “partner support” (factor 2), and “anticipated structural barriers” (factor 3). Factors were extracted with orthogonal varimax rotated factor loadings above absolute 0.38; Cronbach alpha = 0.77; Kaiser–Meyer–Olkin measure of sampling adequacy = 0.79.

## Discussion

In this study, we used mixed methods approaches to develop and validate a tool to assess readiness to initiate ART among newly diagnosed pregnant women living with HIV (Option B +) in the public health sector in Lusaka, Zambia. We reduced 27 candidate items to 16 using item reduction and extraction of factors methods that included tetrachoric correlation coefficient, Item Response Theory discrimination index as well as factor analysis. Three factors were extracted representing three domains as follows: (1) internalized and anticipated HIV stigma, (2) partner support and (3) anticipated structural barriers.

The main output of this study was the development of a potentially feasible way to measure ‘readiness’ to start ART, which can be a complex construct. In fact, Grimes and Grimes reported in a systematic review that readiness was measured differently by different authors; methods included the trans-theoretical model of Prochaska and DiClemente; Fleury’s motivation theory; readiness as a knowledge based concept and others based readiness on the level of adherence to treatment and care [34]. We considered previous literature and used input from local and international stakeholders in our tool development.

While the benefits of ART in pregnancy for prevention of mother to child transmission have been clearly demonstrated [35], initiating ART in pregnancy has been associated with various challenges ranging from personal factors to structural issues [12]. We earlier reported that about 75% of women started ART on the day of HIV diagnosis and up to 8% had not started by seven days [11]. A study in Uganda found 83% of women who were prescribed ART for option B + felt they were “ready” [36]. They reported that factors affecting ART initiation included desire to stay health and to protect the baby, willingness to start, adequate counseling and knowing the benefits of ART [36]. Although most women eventually do start treatment, delayed treatment is a risk for increased mother to child transmission of HIV. Therefore, such a readiness scale is still necessary to identify the women who are not ready to start ART immediately or soon after HIV diagnosis. We believe this is necessary in order to have efficient allocation of scarce resources needed to offer additional counseling and home visit support. Another study in Lusaka, Zambia, found that only 77.8% of pregnant women living with HIV reported willingness to initiate life-long ART. Good adherence, however, has been reported in the same setting [37]. While good adherence is a prerequisite for achieving elimination of mother to child transmission of HIV transmission, this must be matched with adequate ART coverage. Achieving good ART coverage requires resources to provide personalized counselling and follow up to women not ready to initiate ART immediately or soon after HIV diagnosis. The challenge of identifying the woman living with HIV who needs extra support to initiate and remain on ART remains a hindrance to prioritizing the limited resources available in HIV care. The findings in this study highlight the main areas that need to be addressed in the assessment of ART readiness in pregnant women living with HIV in the option B + setting. The use of a readiness assessment tool that could be administered by a lay worker would be a starting point for targeted support.

Our final tool, which encompasses 16 items across several areas, is also supported by other literature as its items have been individually cited as key drivers of engagement in care. A systematic review to assess factors affecting ART initiation, adherence and retention among pregnant and postpartum women living with HIV found that individual, interpersonal, community and structural barriers all affected ART initiation, adherence and/or retention [38]. These findings are similar to the thematic areas we identified as necessary to address when assessing readiness for ART initiation. Stigma has been identified as a barrier to ART initiation by other researchers both in pregnant and non-pregnant populations [39]. Partner support is a critical area that affects a woman’s decision to accept ART. Involvement of a partner in a woman’s care affects disclosure of HIV status which in turn affects ART initiation and ongoing adherence to treatment [12]. Similar to our study findings, structural barriers to ART have been reported by others, including financial challenges, job insecurity, long distance to health facilities and long waiting times due to inadequate numbers of health workers [40, 41].

The main strength of this study was our rigorous approach to design and validate a tool that could be potentially used widely in PMTCT programs in sub-Saharan Africa. Another strength was the collection of data from a relatively large sample size of newly diagnosed pregnant women with HIV. Our study is also innovative in that this is one of few attempts to translate data on HIV care engagement among pregnant women with HIV into a readiness scale [12, 38, 39]. Our study also had several weaknesses. First, we did not perform confirmatory factor analysis to test the dimensionality of the hypothesised factors extracted from the EFA because we lacked a test dataset from either data collected at a different time point in a longitudinal study or a new dataset. Secondly, we did not account for administering the tool in different languages. We acknowledge that this may have resulted in different interpretations by the interviewer or participant; however, we do not believe that this could have significantly affected the results as the two local languages are often spoken by most people interchangeably. Lastly, our data were collected in the early days of option B + ; however, our experience has shown that women accessing PMTCT services still face similar challenges with ART initiation. In addition, studies in similar settings have demonstrated slow uptake of life-long ART among pregnant and breastfeeding women [42] and suboptimal same-day ART initiation among both pregnant and non-pregnant populations [43]. We also acknowledge that the study occurred in urban Zambia and hence the tool could perform differently in other settings, countries or more rural populations. As a next step, we propose to apply the ART readiness tool among newly diagnosed pregnant women living with HIV to further validate it and establish its usefulness.

## Conclusion

We developed and validated a novel tool to measure the construct of ART readiness in pregnant women with newly diagnosed HIV infection in urban Zambia. In a resource limited setting where mother to child transmission remains a public health priority, such a tool if further validated, could be useful to prioritize resources needed to provide extra support for ART initiation and adherence.

## Supplementary Information

**Additional file 1: Table S1.** Tool to assess ‘readiness’ for same day ART initiation among pregnant women living with HIV.

## Data Availability

The data supporting the conclusions of this article are available by submitting a written request to the Director Research, CIDRZ, P.O. Box 34,681, Lusaka, Zambia. This request will be reviewed in consultation with the Principal Investigator.

## Abbreviations

ANC: Antenatal clinic
ART: Antiretroviral therapy
ARV: Antiretroviral drugs
CD4: Cluster of differentiation 4
EFA: Exploratory factor Analysis
FGD: Focus group discussion
HIV: Human immunodeficiency virus
IDI: In-depth interview
IQR: Interquartile range
IRT: Item response theory
KMO: Kaiser–meyer–olkin
NCT: National clinical trial
PMTCT: Prevention of Mother to Child
WHO: World Health Organization
