# Supplementary material for: Development and validation of a novel scale for antiretroviral therapy readiness among pregnant women in urban Zambia with newly diagnosed HIV infection
Source: AIDS Res Ther. 2023 Apr 6;20:21. doi: 10.1186/s12981-023-00509-z (PMC10080880; doi:10.1186/s12981-023-00509-z)
Supplement: Supplementary file 1 — Additional file 1: Table S1. Tool to assess ‘readiness’ for same day ART initiation among pregnant women living with HIV. [file 12981_2023_509_MOESM1_ESM.docx]

**Additional Table S1: Tool to assess ‘readiness’ for same day ART initiation among pregnant women living with HIV**

**Instructions:** Start by informing the patient *“I will read out a series of statements to you. For each, please tell me whether you agree, disagree or are not sure.* Do not read the section titles to the patient as it might prompt their responses in one direction or other. Read out each of the items, and circle the response selected for each item. If needed, say “Do you agree, disagree, or are not sure”. Once you have completed all items, write the numerical score in the column on the right and sum up values to obtain the scores per section and the overall score.

|  |  | | |  |
| --- | --- | --- | --- | --- |
| **Items** | **Disagree** | **Not sure** | **Agree** | **Score** |
| Do you believe that telling someone you have HIV is risky? | 2 | 1 | 0 |  |
| I feel guilty that I have HIV | 2 | 1 | 0 |  |
| Some people who learn my HIV status will start avoiding me | 2 | 1 | 0 |  |
| Some people will tell me that it's my fault I have HIV | 2 | 1 | 0 |  |
| I fear that people who know that I have HIV will tell others | 2 | 1 | 0 |  |
| I worry that people may judge me when they learn my HIV status | 2 | 1 | 0 |  |
| Most people with HIV are rejected when others learn their status  | 2 | 1 | 0 |  |
| TOTAL SCORE FOR SECTION 1  (Internalized and anticipated HIV stigma) |  | | |  |
| If he knew my HIV status, my partner would help me with taking ARVs | 0 | 1 | 2 |  |
| If he knew my HIV status, my partner would leave me or chase me from the house | 2 | 1 | 0 |  |
| My partner cares about me and takes care of me | 0 | 1 | 2 |  |
| My partner would escort me to the clinic if I asked | 0 | 1 | 2 |  |
| Would knowing your partner's status encourage you to take your ARVs? | 2 | 1 | 0 |  |
| TOTAL SCORE FOR SECTION 2  (Partner support) |  | | |  |
| I am afraid that ARVs will cause serious side effects | 2 | 1 | 0 |  |
| I fear that i may not always have enough food to take with my ARVs | 2 | 1 | 0 |  |
| The cost of transportation to clinic may keep me from picking up my refills of ARVs on time | 2 | 1 | 0 |  |
| It will be hard to find time to come to clinic to pick up my ARVs | 2 | 1 | 0 |  |
| TOTAL SCORE FOR SECTION 3  (Anticipated structural barriers) |  | | |  |
| **TOTAL OVERALL SCORE** |  | | |  |
